# Comparison of neural substrates of temporal discounting between youth with autism spectrum disorder and with obsessive-compulsive disorder

**DOI:** 10.1017/S0033291717001088

**Published:** 2017-04-24

**Authors:** C. O. Carlisi, L. Norman, C. M. Murphy, A. Christakou, K. Chantiluke, V. Giampietro, A. Simmons, M. Brammer, D. G. Murphy, MRC AIMS consortium, D. Mataix-Cols, K. Rubia

**Affiliations:** 1 Department of Child and Adolescent Psychiatry, Institute of Psychiatry, Psychology and Neuroscience, King's College, London, UK; 2 Department of Forensic and Neurodevelopmental Sciences, Sackler Institute for Translational Neurodevelopmental Sciences, Institute of Psychiatry, Psychology and Neuroscience, King's College, London, UK; 3 Behavioural Genetics Clinic, Adult Autism Service, Behavioural and Developmental Psychiatry Clinical Academic Group, South London and Maudsley Foundation NHS Trust, London, UK; 4 Centre for Integrative Neuroscience and Neurodynamics, School of Psychology and Clinical Language Sciences, University of Reading, Reading, UK; 5 Department of Neuroimaging, Institute of Psychiatry, Psychology and Neuroscience, King's College, London, UK; 6 National Institute for Health Research (NIHR) Biomedical Research Centre (BRC) for Mental Health at South London and Maudsley NHS Foundation Trust and Institute of Psychiatry, Psychology & Neuroscience, King's College London, London, UK; 7 Department of Neurobiology, Care Sciences and Society, Center for Alzheimer Research, Division of Clinical Geriatrics, Karolinska Institutet, Stockholm, Sweden; 8 Department of Clinical Neuroscience, Centre for Psychiatry Research, Karolinska Institutet, Stockholm, Sweden

**Keywords:** ASD, fMRI, OCD, temporal discounting

## Abstract

**Background:**

Autism spectrum disorder (ASD) and obsessive-compulsive disorder (OCD) share abnormalities in hot executive functions such as reward-based decision-making, as measured in the temporal discounting task (TD). No studies, however, have directly compared these disorders to investigate common/distinct neural profiles underlying such abnormalities. We wanted to test whether reward-based decision-making is a shared transdiagnostic feature of both disorders with similar neurofunctional substrates or whether it is a shared phenotype with disorder-differential neurofunctional underpinnings.

**Methods:**

Age and IQ-matched boys with ASD (*N* = 20), with OCD (*N* = 20) and 20 healthy controls, performed an individually-adjusted functional magnetic resonance imaging (fMRI) TD task. Brain activation and performance were compared between groups.

**Results:**

Boys with ASD showed greater choice-impulsivity than OCD and control boys. Whole-brain between-group comparison revealed shared reductions in ASD and OCD relative to control boys for delayed-immediate choices in right ventromedial/lateral orbitofrontal cortex extending into medial/inferior prefrontal cortex, and in cerebellum, posterior cingulate and precuneus. For immediate-delayed choices, patients relative to controls showed reduced activation in anterior cingulate/ventromedial prefrontal cortex reaching into left caudate, which, at a trend level, was more decreased in ASD than OCD patients, and in bilateral temporal and inferior parietal regions.

**Conclusions:**

This first fMRI comparison between youth with ASD and with OCD, using a reward-based decision-making task, shows predominantly shared neurofunctional abnormalities during TD in key ventromedial, orbital- and inferior fronto-striatal, temporo-parietal and cerebellar regions of temporal foresight and reward processing, suggesting trans-diagnostic neurofunctional deficits.

## Introduction

Autism Spectrum Disorder (ASD) is characterized by social communication difficulties and stereotyped repetitive behaviours (American Psychiatric Association, 2013) with a prevalence of 0.6–2%, predominantly in males (Blumberg *et al.* 2013). Obsessive-Compulsive Disorder (OCD) involves recurrent, intrusive and distressing thoughts (obsessions) and repetitive rituals (compulsions) (American Psychiatric Association, 2013), affecting 1–3% of the population with a higher male prevalence in children (Ruscio *et al.* 2010). These disorders are highly comorbid, with rates exceeding 30% (Simonoff *et al.* 2008) and can sometimes be clinically difficult to separate (Doshi-Velez *et al.* 2014).

The allowance of co-diagnosis of OCD with ASD in DSM-5 questions whether phenotypes common to both disorders are mediated by shared or disorder-specific mechanisms. Characteristic behaviours observed in ASD are wide-ranging and heterogeneous but can include physical rocking, tapping, counting and behavioural inflexibility (e.g. insistence on performing actions in a certain order). Similarly, behaviours in OCD vary widely, but compulsions often include hand-washing, checking, and, sometimes seemingly similar to ASD, counting and behavioural inflexibility surrounding order and symmetry. It has been hypothesized that in both cases, these behaviours may relate to abnormalities in fronto-striatal circuitry that is also important in reward-based decision-making (Langen *et al.* 2011). In ASD, repetitive behaviours are often considered soothing and rewarding, while in OCD, compulsions are performed to reduce anxiety and are often debilitating. However, despite this distinction, converging evidence suggests repetitive behaviours in ASD and OCD may be mediated by shared mechanisms including behavioural disinhibition or motivation control (Hollander *et al.* 2007). Such impairments may maintain diminished control over repetitive behaviours in ASD and compulsions in OCD and involve goal-directed reward-based decision-making. A meta-analysis of structural and functional neuroimaging studies comparing ASD and OCD found shared reduced structure and function during cognitive control in medial prefrontal regions but that OCD had disorder-specific increased function and structure in basal ganglia and insula while ASD had disorder-specific functional reduction in DLPFC and reduced PCC deactivation, presumably reflecting disorder-specific fronto-striato-insular dysregulation in OCD but fronto-striato-insular maldevelopment in ASD, both underpinned by shared reduced prefrontal control (Carlisi *et al.* 2016*b*).

Both disorders also share deficits in motivated ‘hot’ executive functions (EF) (Zelazo & Müller, 2007) including reward-based decision-making measured by choice-impulsivity tasks of gambling and temporal discounting (TD) (Hill, 2004; Sanders *et al.* 2008; Abramovitch *et al.* 2013; Chen *et al.* 2016). TD requires choosing between small immediate rewards and larger later rewards, assessing the extent to which a reward is subjectively discounted when delayed in time (Rubia *et al.* 2009). The ability to inhibit immediate reward choices and wait for larger rewards depends on well-developed frontal lobe-mediated motivation control and temporal foresight and is a key for mature decision-making. A TD function is typically hyperbolic, with steeper rates reflecting more impulsive choice behaviour (Richards *et al.* 1999) (see online Supplement). TD matures with age (Christakou *et al.* 2011; Steinbeis *et al.* 2016) and varies among individuals (Odum, 2011), with steeper TD observed in younger people and individuals with attention deficit hyperactivity disorder (ADHD) and related impulsive disorders (Rubia *et al.* 2009; Noreika *et al.* 2013). Functional magnetic resonance imaging (fMRI) studies of TD in healthy adults and children implicate ventromedial-fronto-limbic networks of reward-based decision-making and dorsolateral and inferior-fronto-insula-striato-parietal networks of temporal foresight (Christakou *et al.* 2011; Chantiluke *et al.* 2014*b*; Wesley & Bickel, 2014).

People with ASD have been shown to have deficits in reward-motivated and forward-thinking behaviour including reward processing and reversal learning (Scott-Van Zeeland *et al.* 2010; Chantiluke *et al.* 2015*a*), incentive processing (Dichter *et al.* 2012), planning (Ozonoff & Jensen, 1999; Geurts *et al.* 2004; Hill, 2004) and TD (Chantiluke *et al.* 2014*b*). However, there have also been negative findings (Antrop *et al.* 2006; Demurie *et al.* 2013). ASD is characterized by fronto-temporo-limbic abnormalities mediating socio-emotional processes (Via *et al.* 2011; Philip *et al.* 2012; Carlisi *et al.* 2016*b*), and in ventromedial/fronto-limbic brain regions involved in TD (Christakou *et al.* 2011; Peters & Büchel, 2011) during reward-related and planning tasks (Just *et al.* 2007; Schmitz *et al.* 2008; Dichter *et al.* 2012; Kohls *et al.* 2013). However, only one fMRI study has been published investigating the neural correlates of TD in adolescents with ASD, which found a weaker relationship between task-performance and bilateral superior temporal and right insular activation relative to controls (Chantiluke *et al.* 2014*b*).

Patients with OCD show deficits during planning (van den Heuvel *et al.* 2011; Shin *et al.* 2014), goal-directed learning (Gillan & Robbins, 2014; Voon *et al.* 2015), reward-based decision-making, gambling (Grassi *et al.* 2015; Figee *et al.* 2016), and incentive processing (Figee *et al.* 2011). Despite evidence that heightened impulsivity is a phenotype associated with OCD (Benatti *et al.* 2014), only one (Sohn *et al.* 2014) of three TD studies in OCD (Vloet *et al.* 2010; Pinto *et al.* 2014; Sohn *et al.* 2014) found performance deficits.

Neuroimaging studies show that OCD is characterized by structural and functional abnormalities in medial and orbitofronto-striato-thalamo-cortical networks mediating EF (Menzies *et al.* 2008; Radua *et al.* 2010; Carlisi *et al.* 2016*b*; Norman *et al.* 2016). No fMRI studies, however, have investigated TD in OCD. Studies using other decision-making tasks in OCD have found hyperactivity in ventral-affective regions including ventromedial prefrontal, orbitofrontal and rostral anterior cingulate cortex (rACC) projecting to ventral striatum and mediodorsal thalamus, and hypoactivity in dorsal-cognitive cortico-striato-thalamic regions including dorsolateral prefrontal (DLPFC), temporal and parietal association cortex projecting to the dorsal striatum and caudate in patients relative to controls (Menzies *et al.* 2008; Brem *et al.* 2012). Hypoactivation in DLPFC and caudate has furthermore been shown in OCD patients during planning (van den Heuvel *et al.* 2005, 2011).

This suggests that ASD and OCD have abnormalities during planning and ‘hot’ EF tasks including reward-based decision-making, and that this may be underpinned by ventromedial and dorsolateral prefronto-striato-limbic abnormalities. However, it is unclear whether reward-based decision-making problems in both disorders are underpinned by shared trans-diagnostic mechanisms or by disorder-specific underlying abnormalities.

We hypothesized that adolescents with ASD would be more impaired on TD relative to adolescents with OCD and controls (Scott-Van Zeeland *et al.* 2010; Chantiluke *et al.* 2014*b*; Chen *et al.* 2016) and that both clinical groups compared with healthy controls would show underactivation in underlying ventromedial prefrontal, limbic and striatal regions mediating TD (Fineberg *et al.* 2009), reflecting a trans-diagnostic neurofunctional phenotype (Chantiluke *et al.* 2015*a*; Grassi *et al.* 2015; Chen *et al.* 2016). However, we hypothesized that people with OCD would show disorder-specific (ventro)medial and dorsolateral-prefrontal dysfunction (Menzies *et al.* 2008; Carlisi *et al.* 2016*b*; Norman *et al.* 2016) while ASD adolescents would show disorder-specific insular and temporo-parietal dysfunction compared to controls (Di Martino *et al.* 2009; Chantiluke *et al.* 2014*b*; Carlisi *et al.* 2016*b*).

## Methods

### Participants

Sixty-nine right-handed (Oldfield, 1971) boys (20 controls, 29 boys with ASD, 20 boys with OCD), 11–17 years, IQ ⩾ 70 (Wechsler, 1999) participated. Medication–naïve boys with high-functioning ASD were recruited from local clinics and support-groups. ASD diagnosis was made by a consultant psychiatrist using ICD-10 research diagnostic criteria (WHO, 1992) and confirmed with the Autism Diagnostic Interview-Revised [ADI-R; (Lord *et al*. 1994)]. The ADI-R and the Autism Diagnostic Observation Schedule [ADOS; (Lord *et al.* 2000)] were completed for all ASD boys; all 29 reached autism cut-offs on all ADI-R (social/communication/restricted/stereotyped) and ADOS (communication/social) domains. ASD participants either fulfilled ICD-10 research diagnostic criteria for autism (*N* = 7) or fulfilled these criteria but had no history of language delay and therefore were subtyped with Asperger's syndrome (*N* = 22). Parents of ASD boys completed the Social Communication Questionnaire [SCQ; (Rutter *et al.* 2003)] and the Strengths and Difficulties Questionnaire [SDQ; (Goodman & Scott, 1999)] (see online Supplement). ASD participants had a physical examination to exclude comorbid medical disorders and biochemical, haematological and chromosomal abnormalities associated with ASD. None of the ASD individuals had a comorbid diagnosis of OCD or any psychiatric disorder, and none of the OCD patients had comorbid ASD.

OCD boys were recruited from National and Specialist OCD clinics. Diagnosis was made by a consultant psychiatrist using ICD-10 criteria and confirmed by the Children's Yale-Brown Obsessive-Compulsive Scale [CY-BOCS; (Goodman *et al.* 1989)]. Parents of OCD patients completed the SDQ. Patients with comorbid psychiatric or neurological disorders, including ASD, were not included in the OCD sample, although OCD patients were not specifically assessed for ASD. Four boys were prescribed stable doses of antidepressants (see online Supplement).

Twenty age and handedness-matched healthy controls were recruited locally by advertisement. Controls scored below clinical threshold on the SDQ and SCQ for any disorder and did not have any psychiatric condition.

Exclusion criteria for all participants included comorbid psychiatric or medical disorders affecting brain development (e.g. epilepsy/psychosis), drug/alcohol dependency, head injury, genetic conditions associated with ASD, abnormal structural brain scan and MRI contraindications. All controls also participated in previously published studies testing fluoxetine effects on TD in ADHD (Carlisi *et al.* 2016*a*) and neurofunctional maturation of TD in healthy adults and adolescents (Christakou *et al.* 2011); all but four ASD boys participated in our fMRI TD study comparing ASD and ADHD (Chantiluke *et al.* 2014*b*). Most ASD and control participants also participated in other fMRI tasks during their visit, published elsewhere (Christakou *et al.* 2013*a*, 2013*b*; Chantiluke *et al.* 2014*a*, 2015*a*, *b*; Murphy *et al.* 2014).

This study was conducted in accordance with the Declaration of Helsinki. Ethical approval was obtained from the local Research Ethics Committee (05/Q0706/275). Study details were explained to child and guardian, and written informed consent was obtained for all participants.

### TD paradigm

**Fig. 1.**
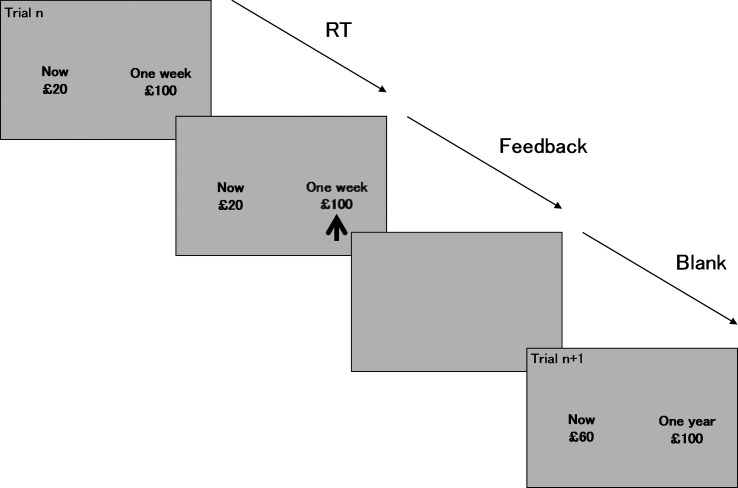
Schematic of the temporal discounting fMRI paradigm. Subjects are asked to indicate whether they would prefer a small, variable amount of money immediately (immediate reward), or whether they would rather wait for a larger delay (up to £100) later (delayed reward). An algorithm adjusts the amount of the immediate reward offered based on the choices of the participant, so as to determine the lowest immediate reward they would tolerate before instead choosing to wait for the larger delayed reward. Three hypothetical delays are presented in random order: 1 week, 1 month and 1 year. Each delay choice is presented 20 times. Trials start with the presentation of the choice display, which remains available for 4 s, within which the subject must choose between the immediate (always on left side) and delayed (always on right) rewards. Total trial duration is 12 s.

Prior to scanning, subjects practiced the 12-min TD task (Rubia *et al.* 2009; Christakou *et al.* 2011; Chantiluke *et al.* 2014*b*) in a mock-scanner. Subjects chose by pressing a left/right button with right index/middle-finger between receiving a small amount of money immediately (£0-£100) or receiving £100 in 1 week, month or year (Fig. 1). Delays (20 trials each) were randomized, but the delayed option (£100) was consistently displayed on the right side of the screen, and variable immediate choices on the left, minimizing sensorimotor mapping effects. Choices were displayed for 4 s, followed by a blank screen of at least 8 s (inter-trial-interval:12 s). The immediate reward amount was adjusted through an algorithm based on previous choices and calculated separately for each delay. This narrows the range of values, converging on an indifference point where the immediate reward is subjectively considered equivalent to the delayed amount for the given delay (Rubia *et al.* 2009), ensuring comparable numbers of immediate and delayed choices for analysis.

### Analysis of performance data

To estimate TD steepness for each subject, indifference values between the immediate amount and delayed £100 for each delay were calculated, equal to the participant's subjective value of £100 after each delay and defined as the midpoint between the lowest chosen immediate reward and the next lowest immediate reward available (i.e. the value of the immediate reward offered at which point the subject began to choose the delayed reward) (Christakou *et al.* 2011).

TD was measured using area under the curve (AUC) (Myerson *et al.* 2001). Smaller AUC denotes steeper discounting rates (i.e. increased choice-impulsivity) (see online Supplement).

One-way between-group analysis of variance (ANOVA) was conducted with AUC as dependent measure to examine group-differences.

### fMRI image acquisition

Gradient-echo echo-planar imaging (EPI) data were acquired at King's College London on a 3T-General Electric SIGNA HDx MRI scanner (Milwaukee, WI) using the body coil for radio frequency transmission and a quadrature birdcage head coil for reception. See online Supplement for acquisition parameters. Total scan was 1.5 h during which subjects completed 2–3 additional fMRI tasks.

### fMRI image analysis

Event-related data were acquired in randomized trial presentation and analysed using the non-parametric XBAM package (v4.1) [www.brainmap.co.uk; (Brammer *et al.* 1997)]. The individual and group-level analysis methods are described in detail elsewhere (Brammer *et al.* 1997; Bullmore *et al.* 1999*b*; Cubillo *et al.* 2014) and in the online Supplement.

Briefly, fMRI data were realigned to minimize motion-related artefacts and smoothed using a 7.2 mm full-width-at-half-maximum (FWHM) Gaussian filter (Bullmore *et al.* 1999*a*). Time-series analysis of individual activation was performed with a wavelet-based resampling method (Bullmore *et al.* 2001). The main experimental conditions were convolved with 2 Poisson model functions (peaking at 4 and 8 s). The weighted sum of these convolutions giving the best fit (least-squares) to the time series at each voxel was calculated. A goodness-of-fit statistic (SSQ ratio) was then computed at each voxel consisting of the ratio of the sum of squares of deviations from the mean intensity value due to the model (fitted time series) divided by that of the squares due to the residuals (original minus model time series). This statistic, the SSQ ratio, was used in further analyses. Individual maps were then normalised to Talairach space (Talairach & Tournoux, 1988), and a group activation map was produced for each group.

#### ANCOVA of between-group effects

One-way between-group analysis of covariance (ANCOVA) with age as covariate was conducted using randomization-based testing to investigate case-control differences (Bullmore *et al.* 1999*b*, 2001). For these comparisons, statistical thresholds of 0.05 (voxel-level)/0.015 (cluster-level) were selected to obtain <1 false-positive 3D cluster per map. Standardized blood-oxygenation level-dependent (BOLD) responses were extracted from significant clusters for each participant and plotted to determine effect direction. *Post*-*hoc* significance was determined among pairwise comparisons using a one-way ANOVA.

#### Influence of behaviour, symptoms and medication

To examine whether clusters showing significant group effects were related to TD performance or symptoms, BOLD response from these clusters was extracted for each participant and Spearman correlations (two-tailed) were performed with AUC and symptom subscales within each group. FMRI analyses were also repeated including AUC as covariate.

Lastly, analyses were repeated excluding the four OCD participants prescribed medication.

## Results

### Participants

There were no significant group-differences in age and IQ (Table 1). Multivariate ANOVAs showed group-differences on SDQ scores; *Post-hoc* tests revealed that patients had higher total-scores than controls, with ASD being more impaired than OCD patients (all *p* < 0.001). On the emotional-distress subscale, both patient groups were more impaired than controls (*p* < 0.001) but did not differ from each other. On all other SDQ subscales, ASD patients were significantly more impaired than controls and OCD patients (all *p* < 0.005), who did not differ on any measure, with the exception of the conduct subscale where ASD patients differed from controls only (*p* < 0.001).

**Table 1.** Participant characteristics for healthy control boys and patients with OCD or ASD

Variables	HC (*N* = 20) Mean (s.d.)	ASD (*N* = 29) Mean (s.d.)	OCD (*N* = 20) Mean (s.d.)	*F* test (df)	*p* value
Age (years)	15.29 (1.8)	14.72 (1.8)	15.74 (1.4)	2.22 (2,66)	0.12
IQ	118.90 (11.9)	113.17 (13.1)	117.70 (13.4)	1.38 (2,66)	0.26
SCQ total score	2.32 (2.3)	18.66 (8.1)	−	76.98 (1,47)	<0.001
SDQ total score	5.58 (4.2)	19.66 (6.8)	12.45 (5.6)	35.56 (2,66)	<0.001
SDQ emotional distress subscale	0.93 (1.8)	4.38 (2.9)	4.35 (2.6)	13.12 (2,66)	<0.001
SDQ conduct subscale	0.86 (1.1)	2.69 (2.2)	1.85 (1.5)	6.55 (2,66)	0.003
SDQ peer relations subscale	1.53 (1.7)	6.59 (2.3)	3.30 (3.0)	28.72 (2,66)	<0.001
SDQ hyperactive impulsive/inattentive subscale	2.72 (2.4)	5.93 (2.6)	2.95 (2.7)	12.52 (2,66)	<0.001
SDQ prosocial behaviour subscale	8.38 (2.4)	4.41 (2.4)	7.65 (2.6)	18.61 (2,66)	<0.001
ADOS communication score	–	3.62 (1.2)	–	–	–
ADOS social interaction score	–	9.03 (2.3)	–	–	–
ADOS communication + social	–	12.66 (3.1)	–	–	–
ADOS stereotypy score	–	1.52 (1.5)	–	–	–
ADI communication score	–	16.59 (4.7)	–	–	–
ADI social interaction score	–	19.97 (5.3)	–	–	–
ADI repetitive behaviour score	–	6.45 (2.4)	–	–	–
CY-BOCS total score	–	–	22.33 (5.8)	–	–
CY-BOCS – obsessions	–	–	10.79 (3.6)	–	–
CY-BOCS – compulsions	–	–	12.01 (3.1)	–	–

ADI, autism diagnostic interview; ADOS, autism diagnostic observation schedule; ASD, autism spectrum disorder; CY-BOCS, Children's Yale-Brown obsessive-compulsive symptoms checklist; HC, healthy controls; OCD, obsessive-compulsive disorder; SCQ, social communication questionnaire; SDQ, strengths and difficulties questionnaire.

### Performance

AUC correlated inversely with *k* (as measured by the square-root transform of these values: *r* = −0.555, *p* < 0.001), suggesting adequate congruency between these two metrics. AUC differed between groups [controls: 0.56 ± 0.13; ASD: 0.45 ± 0.24; OCD: 0.59 ± 0.15; *F*(2,66) = 4.04, *p* = 0.02]. *Post-hoc* comparisons showed that ASD patients had significantly smaller AUC compared with controls (*p* < 0.05) and OCD patients (*p* < 0.01), indicating ASD patients discounted rewards more steeply than the other groups, who did not differ from each other.

### fMRI data

#### Movement

Multivariate ANOVA showed no group-differences in mean head rotation [*F*(2,66) = 1.17, *p* = n.s.] or translation [*F*(2,66) = 2.59, *p* = n.s.] in 3-dimensional Euclidian space.

#### Group maps of brain activation for delayed-immediate choices

See online Supplement for maps of brain activation within each group for the contrast of delayed-immediate choices (online Supplementary Fig. S1).

#### Group-effects on brain activation

**Fig. 2.**
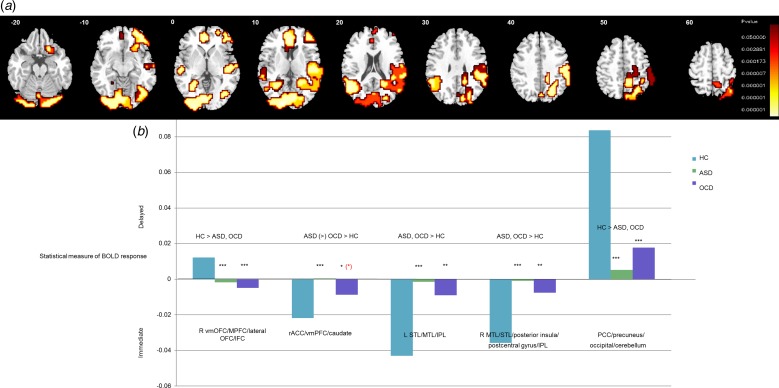
Between-group activation differences for delayed minus immediate choices. (*a*) Axial slices showing split-plot analysis of variance (ANOVA) effects of group on brain activation to delayed – immediate choices. Talairach *Z* coordinates are indicated for slice distance (in mm) from the intercommissural line. The right side of the image corresponds to the right side of the brain. (*b*) Extracted statistical measures of BOLD response are shown for each of the three groups for each of the brain regions that showed a significant group effect. Black asterisks indicate a significant difference between controls and patient group. Red asterisk indicates a difference between the two patient groups. (*) = significant at a trend level; * = significant at the *p* < 0.05 level; ** = significant at the *p* ⩽ 0.005 level; *** = significant at the *p* ⩽ 0.001 level.

One-way ANOVA showed a significant group-effect for delayed-immediate choices in right ventromedial orbitofrontal cortex (vmOFC) extending into MPFC/lateral OFC/inferior frontal cortex (IFC), in cerebellum extending into occipital lobe/posterior cingulate (PCC)/precuneus, in rACC/vmPFC extending into left caudate, in left superior/middle temporal lobe (STL/MTL)/inferior parietal lobe (IPL) and in right MTL/STL extending into posterior insula/postcentral gyrus/IPL (Fig. 2*a*; Table 2). ANCOVA including AUC as covariate showed that effects in rACC/vmPFC and PCC/precuneus were related to task performance.

**Table 2.** Between-group activation differences for delayed minus immediate choices

Brain regions of activation difference	Brodmann area (BA)	Peak Talairach coordinates (*x*, *y*, *z*)	Voxels	Cluster *p* value
(A) HC > OCD, ASD				
R vmOFC/MPFC/lateral OFC/IFC	47/11/25/10/46	40, 56, −13	189	0.009
PCC/precuneus/occipital lobe/cerebellum	31/7/19/18/17	−14, −89, 4	1060	0.0003
(B) OCD, ASD > HC				
rACC/vmPFC/left caudate	10/32/24	0, 41, 4	137	0.01
L STL/MTL/IPL	22/39/40/7/19	−51, −56, 9	273	0.005
R MTL/STL/posterior insula/postcentral gyrus/IPL	22/39/19/5/3/1/2/4/40/7	61, −22, 9	654	0.001

ASD, autism spectrum disorder; HC, healthy controls; IFC, inferior frontal cortex; IPL, inferior parietal lobe; L, left; MTL, middle temporal lobe; OCD, obsessive-compulsive disorder; OFC, orbitofrontal cortex; R, right; STL, superior temporal lobe; rACC, rostral anterior cingulate cortex; vmOFC, ventromedial orbitofrontal cortex; vmPFC, ventromedial prefrontal cortex.

*Post-hoc* analyses based on extracted SSQs showed that abnormalities in vmOFC/MPFC/IFC were shared between OCD and ASD patients, who had increased activation to immediate-delayed choices relative to controls (both *p* < 0.001), who had more activation to delayed choices. In cerebellum/occipital lobe/PCC/precuneus, ASD and OCD patients had reduced activation to delayed-immediate choices compared with controls (both *p* < 0.001). In rACC/vmPFC/caudate, both patient groups had decreased activation to immediate-delayed choices relative to controls (ASD*: p* < 0.001; OCD: *p* < 0.05), who had enhanced activation to immediate-delayed choices, but this effect was more pronounced in ASD *v*. OCD patients at trend-level (*p* < 0.1). Findings in right MTL/STL/insula/postcentral gyrus/IPL (all *p* < 0.005) and left STL/MTL/IPL were due to shared abnormalities in ASD (*p* < 0.001) and OCD (*p* < 0.005) patients, who had less activation to immediate-delayed choices relative to controls who activated this region for immediate *v*. delayed choices (Fig. 2*b*). When the four OCD patients prescribed medication were excluded from analyses, main findings remained, suggesting medication did not influence task-related activation.

#### Correlations between differentially activated brain regions and performance

Correlations between areas that differed between groups and AUC showed that greater activation to delayed-immediate choices in cerebellum/occipital lobe/PCC/precuneus was correlated with less-steep TD in the ASD (*r* = 0.66, *p* < 0.001) and OCD groups (*r* = 0.45, *p* *<* *0*.05). Greater activation to immediate-delayed choices in left STL/IPL correlated with less-steep TD performance in the ASD group (*r* = −0.41, *p* *<* *0*.05). In right MTL/STL/insula/postcentral gyrus/IPL, it correlated with better TD performance in both ASD (*r* = −0.39, *p* < 0.05) and OCD (*r* = −0.59, *p* < 0.005).

#### Correlations between differentially activated brain regions and symptoms

In ASD boys, greater activation to delayed *v.* immediate choices in right vmOFC/MPFC/lateral OFC/IFC correlated at trend-level with lower symptom severity on the repetitive behaviour subscale of the ADI-R (*r* = −0.34, *p* = 0.07). In bilateral STL/insula, lower repetitive behaviour symptom severity was related to increased activation to immediate-delayed choices in the ASD group (left:*r* = 0.47, *p* < 0.01; right:*r* = 0.42, *p* < 0.05). In the OCD group, increased activation to delayed *v.* immediate choices in cerebellum/occipital lobe/PCC/precuneus correlated with lower symptom severity on the CY-BOCS compulsions subscale (*r* = −0.58, *p* < 0.01). There were no correlations between activation and other subscales from the CY-BOCS in OCD or ADOS/ADI-R in ASD.

## Discussion

This comparison between ASD and OCD adolescents on a ‘hot’ EF measure of decision-making showed disorder-specific impaired TD in ASD relative to OCD boys and controls. Despite this, patients had predominantly shared neurofunctional deficits in key TD areas including vmOFC/MPFC/IFC, bilateral temporo-parietal and cerebellar regions, suggesting that the neural basis of TD is a trans-diagnostic feature of both disorders. In ACC/vmPFC extending into caudate, ASD boys had trend-level more severe underactivation relative to OCD and controls for immediate *v*. delayed choices.

Disorder-specific performance impairment in ASD relative to OCD boys extends previous findings of impairments in ASD during TD (Chantiluke *et al.* 2014*b*), although there have been negative findings (Demurie *et al.* 2012). The absence of performance differences between OCD boys and controls is in line with previous studies (Vloet *et al.* 2010; Pinto *et al.* 2014) [but see (Sohn *et al.* 2014)]. Moreover, ASD boys had elevated scores on the hyperactive-impulsive/inattention subscale of the SDQ compared with OCD boys and controls. The disorder-specific performance impairment in the ASD group may relate to these elevated impulsivity symptoms observed in ASD but not OCD, given that ADHD patients are consistently impaired in TD (Jackson & MacKillop, 2016). This finding exclusive to ASD lends support to the distinction between impulsive and compulsive behaviours (Robbins *et al.* 2012), suggesting that while both disorders exhibit deficits in top-down cognitive control and related circuitry (Dalley *et al.* 2011), ASD individuals exhibit more impulsive decision-making during TD, as evidenced by disorder-specific impairments and possibly supported by trend-level disorder-specific abnormalities in ACC/vmPFC/caudate, while OCD patients are more habitually compulsive, supported by intact choice behaviour and no disorder-specific abnormalities.

Both patient groups had reduced activation relative to controls to delayed-immediate choices in ventromedial and ventrolateral OFC/IFC. Ventromedial and ventrolateral fronto-limbic regions are key temporal foresight areas (Christakou *et al.* 2011; Peters & Büchel, 2011) thought to support calculation of discounted reward value. Moreover, right IFC is a key region for working memory, attention to time and integration of external information with internal value representations, supporting goal-directed EF and mediation of temporal foresight (Wittmann *et al.* 2007; Rubia *et al.* 2009; Carlisi *et al.* 2016*a*) and has previously been shown to be abnormal during reward-related decision-making in both OCD (Bari & Robbins, 2013; Stern & Taylor, 2014) and ASD (Dichter *et al.* 2012; Kohls *et al.* 2013).

Both patient groups showed reduced activation in PCC/precuneus/occipital lobe/cerebellum to delayed-immediate choices compared with controls. These areas are important parts of fronto-limbic-parieto-cerebellar networks involved in motivation, reward evaluation and reward response (Vogt *et al.* 1992; McCoy *et al.* 2003). The cerebellum is typically activated during delayed choices in healthy populations and has been associated with future outcome expectancy and temporal bridging (Smith *et al.* 2003; Wittmann *et al.* 2007, 2010; Rubia *et al.* 2009; Christakou *et al.* 2011; Peters & Büchel, 2011; Noreika *et al.* 2013). We previously found similar effects in ADHD patients relative to controls during the same task, suggesting that cerebellar underactivation maybe a trans-diagnostic feature of disorders that are challenged in TD (Rubia *et al.* 2009). Moreover, given the aforementioned role of fronto-limbic-parieto-temporo-cerebellar networks in motivation and reward evaluation, shared abnormalities in this network could possibly relate to neurofunctional similarities in the motivational and reward salience of e.g. performing repetitive behaviours in each disorder, in line with theories of shared impairments in motivation control underpinning these behaviours in each disorder (Hollander *et al.* 2007). This collectively provides first evidence for shared functional abnormalities in ventromedial and ventrolateral fronto-parieto-striato-cerebellar regions between ASD and OCD.

Conversely, relative to controls, both patient groups had reduced activation to immediate choices in the rACC/vmPFC reaching into caudate. However, these abnormalities were at trend-level more pronounced in ASD relative to OCD, possibly linking to ASD-specific performance impairments. rACC mediates decision conflict (Pochon *et al.* 2008) and typically is increased in activation with decision difficulty during intertemporal choice (Pine *et al.* 2009). Our recent meta-analysis of structural and functional MRI studies also found shared reductions in this region in ASD and OCD relative to controls both in volume and in activation during cognitive control (Carlisi *et al.*, 2016*b*). In this study, however, we find that this dysfunction was trend-wise more impaired in ASD, implying a gradual rather than dichotomic effect of more severe impairment in ASD.

Findings of shared reduced vmPFC, left caudate, posterior insula and STL/IPL activation during immediate *v.* delayed choices in patients relative to controls are in line with a wealth of evidence implicating these regions in temporal foresight and reward-based decision-making as well as possible abnormal maturation of networks mediating these processes in ASD and OCD. We showed previously that vmPFC activation to immediate choices during TD increases with age and AUC, indicating an increase in delay-tolerant behaviour linked to increased limbic-corticostriatal activation with age (Christakou *et al.* 2013*a*). In children and adults, steeper TD has been associated with an imbalance between reduced activation in ventromedial prefrontal and lateral frontal systems mediating evaluation of future reward and temporal foresight, and reduced top-down control over ventral-striatal and limbic systems, which respond to immediate reward (Christakou *et al.* 2011; Peters & Büchel, 2011; Chantiluke *et al.* 2014*b*). Moreover, tasks indexing vmPFC functioning have shown age-dependent increases in sensitivity to future consequences (Crone & van der Molen, 2004) and behavioural control during TD (Steinbeis *et al.* 2016).

The caudate is involved in time discrimination (Smith *et al.* 2003), has been linked to reward expectation and evaluation (Hinvest *et al.* 2011) and is activated during immediate choices in healthy individuals (Christakou *et al.* 2011). In OCD, OFC-caudate loops are proposed to drive impulsivity as well as compulsive behaviour (Fineberg *et al.* 2009; Dalley *et al.* 2011). Thus, results could suggest that adolescents with ASD and OCD both have problems with context-dependent decision-making but that this is more problematic for people with ASD, potentially relating to the findings of disorder-specific behavioural deficits in the ASD group. Moreover, the posterior insula is associated with decision-making in the context of prior risk (Xue *et al.* 2010) and is important for the integration of temporal-affective information (Elliott *et al.* 2000) and temporal encoding (Wittmann *et al.* 2010). While previous studies have found specifically anterior insula activation during TD in children (Rubia *et al.* 2009) and adults (Tanaka *et al.* 2004; Bickel *et al.* 2009; Hinvest *et al.* 2011), the present results highlight a differential abnormality in the posterior insula during reward presentation and internal state evaluation (Elliott *et al.* 2000) shared between ASD and OCD.

Findings of reduced activation to immediate-delayed choices in STL/IPL in ASD relative to controls are in line with evidence of weaker brain-behaviour correlations in this region in ASD relative to controls during TD (Chantiluke *et al.* 2014*b*) and extend these findings to OCD. These regions are important for temporal coding and reward selection (Cardinal, 2006; Christakou *et al.* 2011), suggesting deficits with planning, consistent with behavioural deficits in this domain in ASD (Hill, 2004) and OCD (Shin *et al.* 2014). IPL is specifically sensitive to delay (Rubia *et al.* 1998) and attention-allocation to time (Ortuno *et al.* 2002; Coull, 2004; Rubia, 2006), as well as duration encoding (Wittmann, 2009) and quantity representation, which may contribute to inter-temporal choices regarding the IPL's role in comparing time and value (Sandrini *et al.* 2004). Correlations between enhanced activation to immediate choices in the patient groups and better TD performance suggest that in both groups, this upregulation is related to a shift in performance towards that of controls, providing possible mechanistic implications of this region in the context of TD behaviour. Moreover, increased activation bilaterally in this region in the ASD group correlated with lower levels of repetitive behaviours, linking performance improvement and symptom reduction to brain activation in these individuals, further highlighting the mechanistic implications of this region in the context of repetitive behaviours and decision-making.

Clinically, the fact that these disorders exhibit shared neural abnormalities during TD has implications for identification of common mechanisms, which may drive overlapping behaviours in each disorder. While symptoms such as compulsions in OCD can sometimes appear similar to repetitive behaviours in ASD at an observational level, less is known about the mechanistic underpinnings of these behaviours and related cognitive functions and whether they are shared or disorder-specific. Thus, this evidence sheds light on trans-diagnostic phenotypes that could aid in future treatment targets and work toward providing a biological explanation of commonalities and differences in clinical behaviour. This has similarly been shown in the case of inhibitory control and brain structure/function differences/similarities in a recent meta-analysis comparing ASD and OCD (Carlisi *et al.* 2016*b*), and this study extends this understanding to temporal foresight and decision-making.

This study's strengths include the thoroughness with which ASD individuals were assessed for the presence of ASD-related symptomatology and the exclusion of patients with psychiatric comorbidities. However, sub-threshold symptoms may have been present in the patient samples. The group of ASD patients tested in this study had a relatively high IQ, comparable with that of controls. While matching groups for IQ is important for fMRI studies to disentangle the effects of ASD from the effects of low IQ, this also means that the findings are not generalizable to other more typical ASD patients with low IQ (Charman *et al.* 2011; Crespi, 2016). The fact that most patients had high-functioning Asperger's syndrome further limits generalizability. Thus, it is possible that OCD-related symptoms were present in the ASD sample and could account for some of the neurobiological overlap in results. In addition, sub-clinical levels of ASD-related symptoms may have been present in the OCD sample, as reflected by shared impairments compared with controls on the emotional-distress SDQ subscale. It would also be interesting to examine the possible effects of puberty on any observed abnormalities. However, it has been shown that impulsive behaviour is independent of puberty in males (Steinberg *et al.* 2008). Additionally, four OCD patients were prescribed antidepressant medication. While there is evidence for effects of serotonin on brain function (Murphy *et al.* 2008; Murphy, 2010), results remained when analyses were repeated excluding these patients. Lastly, It is a common finding that brain activation is more sensitive than performance to detect differences between groups in these patient groups (Fitzgerald *et al.* 2010; Duerden *et al.* 2013; Ambrosino *et al.* 2014; Marsh *et al.* 2014; Chantiluke *et al.* 2015*b*; Morein-Zamir *et al.* 2015). While the subject numbers have been shown to be sufficient for fMRI analyses (Thirion *et al.* 2007), the performance and correlation analyses, however, were underpowered.

## Conclusions

This is the first study to compare brain function between these disorders and provides novel evidence to suggest that ASD and OCD share trans-diagnostic abnormalities during TD in ventromedial and ventrolateral fronto-striatal and fronto-temporo-parieto-cerebellar regions important for temporal foresight and reward-related decision-making. This may drive shared problems with reward-related behaviours and delaying repetitive actions.

## Supplementary material

For supplementary material accompanying this paper visit http://dx.doi.org/10.1017/S0033291717001088. click here to view supplementary material

## Declaration Interest

K. R. has received funding from Lilly for another project and speaker's honoraria from Lilly, Shire, Novartis and Medice. D. M. has received funding for another project from Lilly. M. B. has served as a consultant for P1Vital. The other authors have no conflict of interests to declare.
