# Supplementary material for: Comparison of neural substrates of temporal discounting between youth with autism spectrum disorder and with obsessive-compulsive disorder
Source: Psychol Med. 2017 Apr 24;47(14):2513–27. doi: 10.1017/S0033291717001088 (PMC5964452; doi:10.1017/S0033291717001088)
Supplement: Supplementary file 1 [file S0033291717001088sup001.docx]

# Comparison of neural substrates of temporal discounting between youth with Autism Spectrum Disorder and with Obsessive-Compulsive Disorder

# Supplementary information

Christina O Carlisi, BA, Luke Norman, MSc, Clodagh Murphy, MRCPsych, Anastasia Christakou, PhD, Kaylita Chantiluke, PhD, Vincent Giampietro, PhD, Andy Simmons, PhD, Michael Brammer, PhD, Declan Murphy, MD, FRCPsych, the MRC AIMS consortium, David Mataix-Cols, PhD, Katya Rubia, PhD

## METHODS

### Inclusion criteria for patients

All but 5 ASD participants scored above clinical threshold for ASD on the Social Communication Questionnaire (SCQ; ([Rutter *et al.*, 2003](#_ENREF_10))), and those who did not were included on the basis of a clinician-confirmed ASD diagnosis. Eight ASD participants scored above threshold for inattention/hyperactivity problems on the Strengths and Difficulties Questionnaire (SDQ; ([Goodman and Scott, 1999](#_ENREF_6))), but these patients were not excluded on the basis that inattention problems are common in ASD and clinician confirmation that ASD symptoms were the sole/primary clinical concern for these patients.

One OCD patient scored above clinical cut-off for inattention/hyperactivity symptoms on the SDQ subscale, but this participant was not excluded on the basis that communication and inattention difficulties can be conflated with symptoms related to OCD and the fact that no OCD patients met criteria for ASD or ADHD based on clinical interview.

### OCD patient medication status

Patient 1: Sertraline 75mg

Patient 2: Sertraline 100mg

Patient 3: Sertraline 200mg

Patient 4: Fluvoxamine 100mg; risperidone 0.5mg

### TD task

In individually-adjusted TD paradigms ([Christakou *et al.*, 2011](#_ENREF_5), [Richards *et al.*, 1997](#_ENREF_8)), the immediate reward is adjusted using an algorithm based on previous choices of the participant for different delays to narrow the range of immediate values offered for each delay type, converging towards the value of the participant’s subjective equivalent of the fixed delayed reward ([Richards *et al.*, 1999](#_ENREF_9)). This results in the typically hyperbolic delay discounting function.

Reward is typically discounted as a decay function depending on amount, delay and a free impulsiveness indicator *“k”*, calculated by fitting a hyperbolic function to the indifference values for each delay. Written as *V=A/(*1*+kD)*, *V* is the subjective value of reward amount *A*, *D* is the delay and *k* is a constant defining the subject’s rate of discounting, with larger *k* reflecting steeper TD ([Richards *et al.*, 1999](#_ENREF_9)).

However, the limitations of fMRI task adaption such as relatively few trials and only three delay points limits the goodness-of-fit of the data to a non-linear curve function. Additionally, distribution of *k*-values in this study was not normal, skewed by low-frequency high-value outliers. Thus, TD was measured using area under the curve (AUC) ([Myerson *et al.*, 2001](#_ENREF_7)). Normalized subjective values of the delayed £100 for each delay were plotted against the normalized delays, and AUC of these plots were calculated for each participant and used as the main dependent variable.

### fMRI Image Acquisition

In each of 22 non-contiguous planes parallel to the anterior-posterior commissure, 480 T2*-weighted MR images depicting BOLD (blood-oxygen level-dependent) contrasts covering the whole brain were acquired with echo time (TE)=30ms, repetition time (TR)=1.5s, flip angle=60^o^, in-plane voxel size=3.75mm, slice thickness=5mm, slice skip=0.5mm. This EPI dataset provided almost complete brain coverage. A whole-brain high-resolution structural scan (inversion recovery gradient EPI) used for standard space normalization of individual activation maps was acquired in the inter-commissural plane with TE=30ms, TR=3s, flip angle=90^o^, slices=43, slice thickness=3.0mm, slice skip=0.3mm, in-plane voxel-size=1.875mm, providing comprehensive coverage.

### fMRI Data Analysis Methods

#### Individual Analysis

Data were first processed to minimize motion-related artefacts ([Bullmore *et al.*, 1999a](#_ENREF_2)). A 3-D volume consisting of the average intensity at each voxel over the entire experiment was calculated and used as a template. The 3D-image volume at each time point was realigned to this template by computing the combination of rotations (around *x*, *y* and *z* axes) and translations (*x*, *y* and *z*) that maximised the correlation between the image intensities and the volume in question and the template (rigid-body registration). Following realignment, data were smoothed using a Gaussian filter (full-width at half-maximum (FWHM) 7.2mm) to improve the signal-to-noise ratio of the images ([Bullmore *et al.*, 1999a](#_ENREF_2)). Following motion correction, global detrending and spin-excitation history correction, time series analysis for each subject was conducted based on previously published wavelet-based resampling methods for fMRI data ([Bullmore *et al.*, 2001](#_ENREF_3), [Bullmore *et al.*, 1999b](#_ENREF_4)). At the individual-subject level, a standard general linear modelling approach was used to obtain estimates of the response size (beta) to each of the task conditions (delayed and immediate reward choices) against an implicit baseline. We first convolved the main experimental conditions with 2 Poisson model functions (peaking at 4 and 8s). We then calculated the weighted sum of these 2 convolutions that gave the best fit (least-squares) to the time series at each voxel. A goodness-of-fit statistic (SSQ ratio) was then computed at each voxel consisting of the ration of the sum of squares of deviations from the mean intensity value due to the model (fitted time series) divided by that of the squares due to the residuals (original time series minus model time series). The appropriate null distribution for assessing significance of any given SSQ ratio was established using a wavelet-based data re-sampling method ([Bullmore *et al.*, 2001](#_ENREF_3)) and applying the model-fitting process to the resampled data. This process was repeated 20 times at each voxel and the data combined over all voxels, resulting in 20 null parametric maps of SSQ ratios for each subject, which were combined to give the overall null distribution of SSQ ratio. This same permutation strategy was applied at each voxel to preserve spatial correlation structure in the data. Individual SSQ ratio maps were then transformed into standard space, first by rigid-body transformation of the fMRI data into a high-resolution inversion recovery image of the same subject, and then by affine transformation onto a Talairach template ([Talairach and Tournoux, 1988](#_ENREF_11)).

#### Group Analysis

A group-level activation map was produced for each group for the experimental condition (delayed-immediate choices) by calculating the median observed SSQ ratios at each voxel in standard space across all subjects and testing them against the null distribution of median SSQ ratios computed from the identically transformed wavelet-resampled data ([Brammer *et al.*, 1997](#_ENREF_1), [Bullmore *et al.*, 2001](#_ENREF_3)). The voxel-level threshold was first set to 0.05 to give maximum sensitivity and to avoid type-II errors. Next, a cluster-level threshold was computed for the resulting 3D voxel clusters. The necessary combination of voxel and cluster level thresholds was not assumed from theory but rather was determined by direct permutation for each dataset, giving excellent type-II error control ([Bullmore *et al.*, 1999b](#_ENREF_4)). Cluster mass rather than a cluster extent threshold was used to minimize discrimination against possible small, strongly responding foci of activation ([Bullmore *et al.*, 1999b](#_ENREF_4)).

## RESULTS

### fMRI Data – Within-Group Activation Results

#### Controls

For delayed – immediate choices, controls activated left putamen, bilateral insula and temporo-parietal regions including medial and superior temporal lobe and inferior parietal lobe (IPL), as well as posterior cingulate cortex (PCC)/precuneus and pre and post-central gyrus. For immediate – delayed choices, controls activated bilateral cerebellum and occipital lobe, IPL and right pre/post-central gyrus (Fig S1A).

#### ASD patients

For delayed – immediate choices, ASD patients activated left medial temporal lobe and posterior insula, right anterior insula, medial prefrontal cortex (MPFC), PCC/precuneus, and right pre-SMA. For immediate – delayed choices, ASD patients activated bilateral cerebellum and occipital lobe, IPL, right pre/post-central gyrus, and bilateral medial and lateral fronto-striatal regions (Fig S1B).

*OCD patients*

For delayed – immediate choices, OCD patients activated left medial temporal lobe, bilateral middle temporal gyrus, dorsal MPFC (dMPFC) and PCC/precuneus. For immediate – delayed choices, OCD patients activated bilateral cerebellum and occipital lobe, IPL and right pre/post-central gyrus (Fig S1C).

(A) Healthy controls


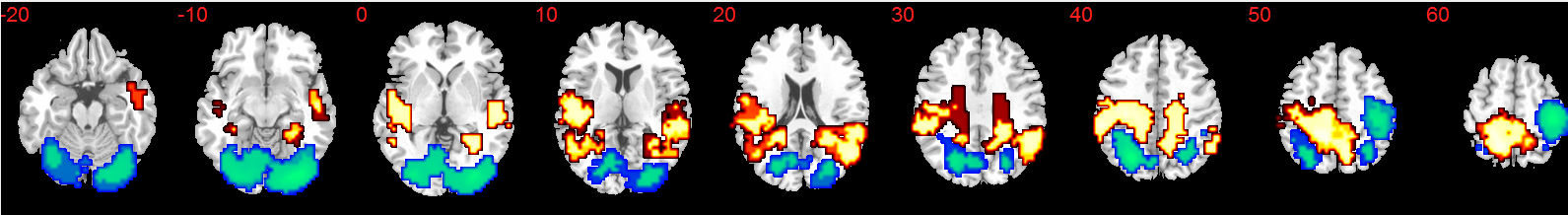


(B) ASD patients


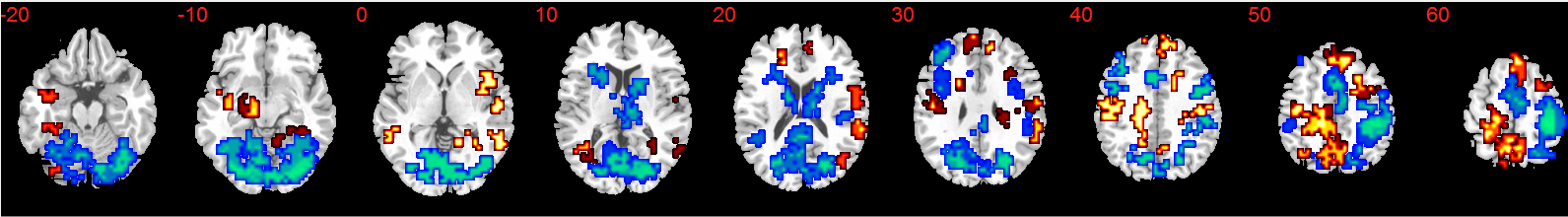


(C) OCD patients


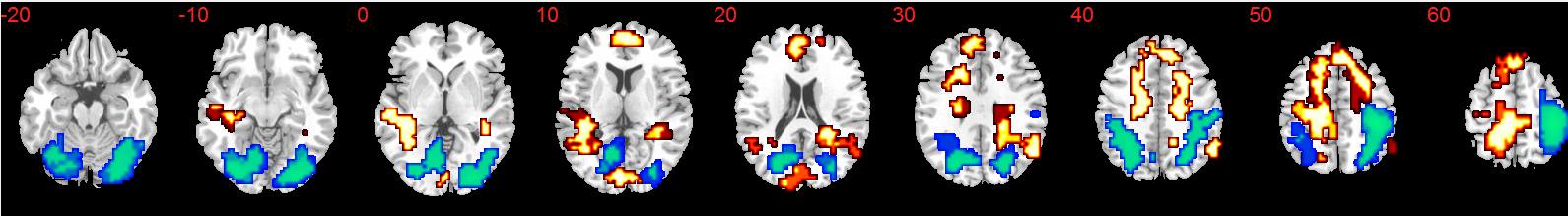


**Supplementary Figure S1.** Group activation maps. Axial slices showing within-group brain activation for the contrasts of delayed-immediate reward choices (red) and immediate-delayed reward choices (blue). (A) Healthy controls, (B) ASD patients and (C) OCD patients. Talairach z-coordinates are indicated for slice distance (in mm) from the intercommissural line. The right side of the brain corresponds to the right side of the image.

References

**Brammer, M. J., Bullmore, E. T., Simmons, A., Williams, S. C. R., Grasby, P. M., Howard, R. J., Woodruff, P. W. R. & Rabe-Hesketh, S.** (1997). Generic brain activation mapping in functional magnetic resonance imaging: A nonparametric approach. *Magnetic Resonance Imaging* **15**, 763-770.

**Bullmore, E., Brammer, M., Rabe-Hesketh, S., Curtis, V., Morris, R., Williams, S., Sharma, T. & McGuire, P.** (1999a). Methods for diagnosis and treatment of stimulus-correlated motion in generic brain activation studies using fMRI. *Human Brain Mapping* **7**, 38-48.

**Bullmore, E., Long, C., Suckling, J., Fadili, J., Calvert, G., Zelaya, F., Carpenter, T. A. & Brammer, M.** (2001). Colored noise and computational inference in neurophysiological (fMRI) time series analysis: resampling methods in time and wavelet domains. *Human Brain Mapping* **12**, 61-78.

**Bullmore, E. T., Suckling, J., Overmeyer, S., Rabe-Hesketh, S., Taylor, E. & Brammer, M. J.** (1999b). Global, voxel, and cluster tests, by theory and permutation, for a difference between two groups of structural MR images of the brain. *Medical Imaging, IEEE Transactions on Medical Imaging* **18**, 32-42.

**Christakou, A., Brammer, M. & Rubia, K.** (2011). Maturation of limbic corticostriatal activation and connectivity associated with developmental changes in temporal discounting. *NeuroImage* **54**, 1344-1354.

**Goodman, R. & Scott, S.** (1999). Comparing the Strengths and Difficulties Questionnaire and the Child Behavior Checklist: Is Small Beautiful? *Journal of Abnormal Child Psychology* **27**, 17-24.

**Myerson, J., Green, L. & Warusawitharana, M.** (2001). Area under the curve as a measure of discounting. *Journal of the Experimental Analysis of Behavior* **76**, 235-243.

**Richards, J. B., Mitchell, S. H., de Wit, H. & Seiden, L. S.** (1997). Determination of discount functions in rats with an adjusting-amount procedure. *Journal of the Experimental Analysis of Behavior* **67**, 353-366.

**Richards, J. B., Zhang, L., Mitchell, S. H. & Wit, H.** (1999). Delay or probability discounting in a model of impulsive behavior: effect of alcohol. *Journal of the Experimental Analysis of Behavior* **71**, 121-143.

**Rutter, M., Bailey, A. & Lord, C.** (2003). *The social communication questionnaire: Manual*. Western Psychological Services.

**Talairach, J. & Tournoux, P.** (1988). *Coplanar stereotaxic atlas of the human brain, a 3-dimensional proportional system: an approach to cerebral imaging*. Thieme: New York.
